# Formulation and Evaluation of Almotriptan Malate Nasal Drops

**Published:** 2009

**Authors:** V. Pradhan, R. Gaikwad, A. Samad, B. Prabhakar

**Affiliations:** School of Pharmacy & Technology Management, SVKM's NMIMS University, V. L. Mehta Road, Vile Parle, Mumbai-400 056, India; 1 Bombay Veterinary College, Department of Nuclear Medicine, Parel, Mumbai-400 012, India

**Keywords:** Nasal solutions, almotriptan malate, nasal residence time, gamma scintigraphy

## Abstract

Nasal solutions of almotriptan malate were prepared in phosphate buffer containing different proportions of HPMC E15. *In vitro* permeation studies were performed using Franz diffusion cell with dialysis membrane and *ex vivo* permeation studies were carried out using sheep nasal mucosal layer. The formulations were radiolabeled with 99mTc and the nasal residence time was studied in rabbits. Nasal irritation was evaluated in rats. Formulations prepared with HPMC E15 5% w/v did not retard the release of almotriptan. Gamma scintigraphy studies showed increased residence time as compared to plain drug solution. No nasal irritation was observed and the formulations were found stable for 3 months.

Migraine is a chronic debilitating disorder which has been rated by WHO amongst the world's top 20 leading causes of disability. Almotriptan malate (AM) is a second generation triptan which has been shown to have efficacy comparable to sumatriptan with an improved tolerability profile[1]. Since migraine is very often associated with nausea and vomiting there is a need for alternate form of delivery to tablets. Nasal drug delivery is attractive since the large surface area of nasal mucosa affords a rapid onset of therapeutic effect and the potential for direct nose to brain delivery. Liquid delivery either as drops or sprays are very popular with patients due to ease of administration. However the mucociliary clearance results in low bioavailability of the drug via nasal route. Several studies are ongoing to improve the efficiency of bio adhesive polymers for nasal drug delivery[2].

## MATERIALS AND METHODS

Almotriptan malate (Enaltec labs), hydroxypropylmethylcellulose (HPMC E15 from Colorcon Asia Ltd.), disodium hydrogen phosphate, sodium dihydrogen ortho phosphate, ^99m^Tc, dialysis membrane, Purified water.

Nasal solutions were prepared containing AM 8.75 mg/0.2 ml, which is equivalent to almotriptan 6.25 mg. Phosphate buffers were used to maintain the solution in the acceptable range of 4.5-6.5. HPMC E15 was added in different proportions from 2.5-7.5% w/v. The osmolarity was recorded on Osmomat 30. Viscosity of the solutions was recorded using a cone and plate viscometer. *In vitro* permeation studies were performed using a Franz diffusion cell with dialysis membrane. The receptor compartment contained simulated nasal fluid (without enzymes) and was maintained at 34±1°. For *ex vivo* studies same procedure was followed except fresh tissues carefully removed from the nasal cavity of sheep obtained from a local slaughter house were used. The percentage of drug release of optimized formulation of AM was fitted in various models such as zero order, first order, Higuchi's release kinetics, Korsmeyer-Peppas equation. The formulations were radiolabeled with ^99m^Tc and administered to rabbits to study the nasal residence time. Nasal irritation study was done on rats to observe tissue irritation to the nasal mucosa after administration of the formulation. The formulations were subjected to stability studies at 25°, 30°/65 % RH and 40°/75% RH.

## RESULTS AND DISCUSSION

The pH of the formulation was kept at 4.5 since the ideal range is 4.5-6.5. The osmolarity of the solution was found to be 405. Viscosity was found to be in the range 0.159-1.818 poise. An antimigraine drug is expected to provide immediate relief to the patient. Mucoadhesives used should not retard the release of the drug. It was observed that formulations prepared with HPMC E15 5% w/v did not retard the release of AM. The release mechanism from the hydrophilic polymers was decided by the “n” value obtained by fitting the data in Korsmeyer-Peppas equation. Gamma scintigraphy studies showed increased residence time as compared to plain drug solution. The nasal tissue showed normal upper respiratory epithelium from cuboidal to columnar appearance. No abnormalities were detected. Formulations were found to be stable up to 3M under accelerated conditions. A further investigation is required to detect the blood and brain levels achieved. AM nasal delivery is a promising alternative to tablets in the treatment of migraine.

**Fig. 1:**
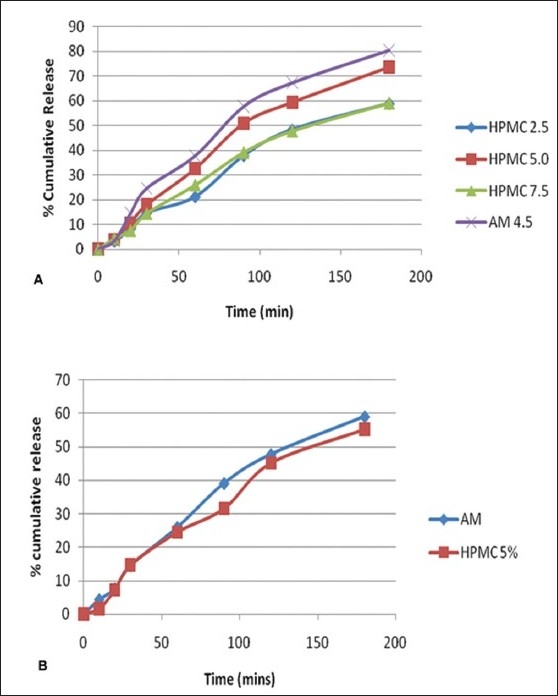
*In vitro* and *ex vivo* permeation studies A. In vitro permeation through dialysis membrane, (-◆-) HPMC 2.5, (-■-) HPMC 5.0, (-▲-) HPMC 7.5 and (-x-) almotriptan malate 4.5 and B. *ex vivo* permeation through sheep nasal mucosa (-◆-) almotriptan malate and (-■-) HPMC 5%

**Fig. 2:**
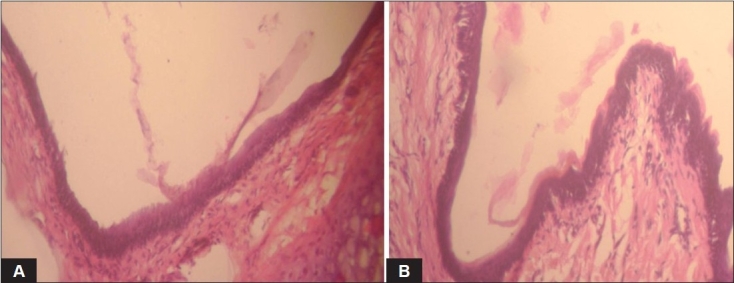
Histopathology of rat nasal mucosa Histopathology of rat nasal mucosa in haematoxylin-eosin stain. A. control sample and B. treated sample
